# Genetic architecture and genomic selection of fatty acid composition predicted by Raman spectroscopy in rainbow trout

**DOI:** 10.1186/s12864-021-08062-7

**Published:** 2021-11-03

**Authors:** Carole Blay, Pierrick Haffray, Jonathan D’Ambrosio, Enora Prado, Nicolas Dechamp, Virginie Nazabal, Jérôme Bugeon, Florian Enez, David Causeur, Christophe Eklouh-Molinier, Vincent Petit, Florence Phocas, Geneviève Corraze, Mathilde Dupont-Nivet

**Affiliations:** 1 Université Paris-Saclay, INRAE, AgroParisTech, GABI, Jouy-en-Josas, France; 2 SYSAAF, Station LPGP-INRAE, Rennes, France; 3 University of Rennes, CNRS, ISCR – UMR 6226, ScanMAT – UMS 2001, Rennes, France; 4 INRAE, LPGP, Rennes, France; 5 Laboratoire de Mathématiques Appliquées, IRMAR, Agrocampus Ouest, Rennes, France; 6 Les Sources de l’Avance, Pissos, France; 7 INRAE, University of Pau & Pays Adour, E2S UPPA, UMR1419 NuMéA, St Pée sur, Nivelle, France

**Keywords:** Fish, Genomic selection, QTL, GWAS, Fatty acid, Raman, Genetic correlations, Accuracy

## Abstract

**Background:**

In response to major challenges regarding the supply and sustainability of marine ingredients in aquafeeds, the aquaculture industry has made a large-scale shift toward plant-based substitutions for fish oil and fish meal. But, this also led to lower levels of healthful n−3 long-chain polyunsaturated fatty acids (PUFAs)—especially eicosapentaenoic (EPA) and docosahexaenoic (DHA) acids—in flesh. One potential solution is to select fish with better abilities to retain or synthesise PUFAs, to increase the efficiency of aquaculture and promote the production of healthier fish products. To this end, we aimed i) to estimate the genetic variability in fatty acid (FA) composition in visceral fat quantified by Raman spectroscopy, with respect to both individual FAs and groups under a feeding regime with limited n-3 PUFAs; ii) to study the genetic and phenotypic correlations between FAs and processing yields- and fat-related traits; iii) to detect QTLs associated with FA composition and identify candidate genes; and iv) to assess the efficiency of genomic selection compared to pedigree-based BLUP selection.

**Results:**

Proportions of the various FAs in fish were indirectly estimated using Raman scattering spectroscopy. Fish were genotyped using the 57 K SNP Axiom™ Trout Genotyping Array. Following quality control, the final analysis contained 29,652 SNPs from 1382 fish. Heritability estimates for traits ranged from 0.03 ± 0.03 (n-3 PUFAs) to 0.24 ± 0.05 (n-6 PUFAs), confirming the potential for genomic selection. n-3 PUFAs are positively correlated to a decrease in fat deposition in the fillet and in the viscera but negatively correlated to body weight. This highlights the potential interest to combine selection on FA and against fat deposition to improve nutritional merit of aquaculture products. Several QTLs were identified for FA composition, containing multiple candidate genes with indirect links to FA metabolism. In particular, one region on Omy1 was associated with n-6 PUFAs, monounsaturated FAs, linoleic acid, and EPA, while a region on Omy7 had effects on n-6 PUFAs, EPA, and linoleic acid. When we compared the effectiveness of breeding programmes based on genomic selection (using a reference population of 1000 individuals related to selection candidates) or on pedigree-based selection, we found that the former yielded increases in selection accuracy of 12 to 120% depending on the FA trait.

**Conclusion:**

This study reveals the polygenic genetic architecture for FA composition in rainbow trout and confirms that genomic selection has potential to improve EPA and DHA proportions in aquaculture species.

**Supplementary Information:**

The online version contains supplementary material available at 10.1186/s12864-021-08062-7.

## Background

Over the last several decades, aquatic food production has evolved away from capture fisheries toward the culture of increasing numbers of farmed fish species [1]. However, aquaculture operations face major challenges regarding the sustainability of the feed used, particularly with regard to marine ingredients. To address these concerns, fish oil and fish meal—the traditional sources of proteins and lipids in aquafeeds—have been largely substituted (60–80%) with plant-based ingredients. However, compared to fish oils, vegetable oils differ in their fatty acid composition: they are rich in oleic acid (OA; C18:1 n-9), linoleic acid (LA; C18:2 n-6), and alpha-linolenic acid (ALA; C18:3 n-3) and contain very low proportion or no n-3 long-chain polyunsaturated fatty acids (n-3 LC PUFAs). In farmed trout, one result of this dietary shift has been reductions in the levels of n-3 LC PUFAs, especially eicosapentaenoic (EPA; C20:5 n-3) and docosahexaenoic (DHA; C22:6 n-3) acids, in flesh [2]. n-3 LC PUFAs are known to have beneficial effects on human health, including the prevention of a range of cardiovascular and inflammatory diseases and neurological disorders [3, 4]. Freshwater fish are theoretically capable of biosynthesising DHA and EPA via desaturation and elongation of the ALA found in some vegetable oils [5]. In practice, however, such bioconversion is insufficient to compensate for the lack of dietary n-3 LC PUFAs, resulting in a significant reduction in levels of these healthful fatty acids (FAs) in fish tissues [5, 6]. Especially, in rainbow trout, two fads2 genes encoding proteins with delta 5 and delta 6 desaturase activities and two elongase enzymes Elovl5 and Elovl 2 have been isolated and functionally characterised [7, 8]. Rainbow trout is dependent on Elovl2 for 22:5 n-3 to 24:5 n-3 synthesis and ultimately DHA synthesis [8]. When diets are high in alpha-linolenic acid (ALA, 18: 3 n-3) with no added EPA or DHA, fads2, Elovl5 and Elovl2 are most highly expressed in rainbow trout liver [9]. PUFAs are also important in the fish life cycle, most notably for their roles in reproduction, egg quality, and offspring development [5], and have effects on nutritional quality of fish flesh for human consumption [6, 10]. The relative amounts of EPA and DHA formed are determined by the activities of desaturase and elongase enzymes, which are themselves influenced by several factors, such as nutrition, the environment, physiology, and genetics [11]. To date, numerous studies have examined the characteristics of FA metabolism and the effect of dietary oil sources on the FA composition of farmed fish in attempts to find solutions for this problem [12–18].

To meet consumer demand, producers are constantly making improvements in husbandry techniques, nutrition, and genetic management. One potential strategy for meeting the demand for farmed fish without compromising nutritional value could be to combine genetic selection with changes in commercial feed formulations. Recent investigations of the genetic variability underlying lipid deposition and metabolism in fish have identified a highly heritable genetic component that governs the capacity to synthesise and/or deposit LC-PUFAs [19–22]. In trout, studies have revealed the efficiency of divergent selection for total fat content [17, 23–25] and potential interactions between dietary lipid level and genetic selection for body fat [26, 27]. Thus, to counteract the diet-based decline in PUFA content in flesh, one solution may be breeding programmes that select fish with better abilities to retain and/or synthesise PUFAs. To date, studies have illustrated the potential of selective breeding to increase n-3 LC PUFA levels in salmon [20, 28–30], yellow croaker [22], tilapia [31], Asian sea bass [32] and common carp [33]. Similar work has also been conducted in trout [30], but as yet, we lack knowledge on the genetic architecture that shapes the relative proportions of individual FAs in this species. A common strategy for this purpose is the genome-wide association study (GWAS), which has been used to identify the genetic regions and loci significantly associated with FA composition in species such as cattle (in meat [34–36] and milk [37]) and pigs [38–41], common carp [42], Asian seabass [32, 43], tilapia [44] and Atlantic salmon [28]. In rainbow trout, though, the genetic parameters of FA composition remain unknown, and no GWAS has been performed to increase our knowledge of the genetics of n-3 LC PUFA composition. Furthermore, the relationships between FA composition and traits related to lipid deposition, weight, yield, or quality have not yet been characterised. Phenotype-based research has shown that total fat content (as measured with a Fatmeter) in the muscle increases with the growth and development of fish [45, 46], but the genetic correlations between production traits and FA composition—which would play a crucial role in optimising the efficiency of breeding programmes—remain unknown.

In the literature, the technology most commonly used for FA characterisation is chemical extraction and gas chromatography. However, such analyses are expensive, invasive, and time-consuming, and are thus not easily applicable to a breeding programme. In this context, what is needed are alternative methods that are affordable and potentially non-invasive, which could be used to estimate the proportions of different FAs (group or individual FA) in a population of fish that is large enough to enable effective analyses of the genetic architecture of traits. One potential approach could be the use of Raman scattering spectroscopy, a rapid, non-destructive method for molecular characterisation based on vibrational spectrometry. Specifically, this method enables the qualitative and quantitative characterisation of molecules through analyses of spectral bands with respect to the fundamental vibrational modes of their chemical bonds. This technology has already been used to determine total lipid concentration in minced salmon flesh [47] and FA composition in pork adipose tissue [48], as well as in fish oils [49, 50] and in Atlantic salmon flesh [51].

**Fig. 1:**
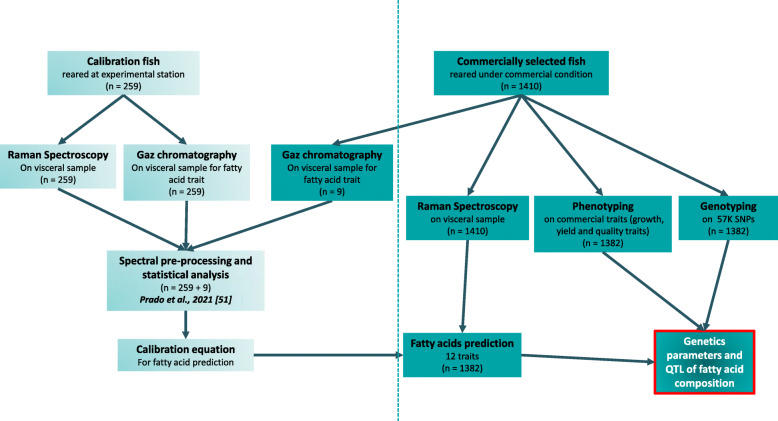
Description of the methodology of the study encompassing gaz chromatography, Raman spectroscopy, phenotyping and genotyping for calibration and commercial selected fish

As a step toward the long-term objective of increasing the nutritional value of farmed rainbow trout and ensuring high levels of n-3 PUFAs in the flesh, the main goal of the present study was to investigate the genetics of n-3 LC PUFA composition. Specifically, we used a commercially selected population of rainbow trout to (1) estimate the genetic variability of the FA composition of visceral adipose tissue, as indirectly estimated by Raman spectroscopy; (2) analyse genetic and phenotypic correlations among different FA traits and between FAs and production traits linked to yields and fat deposition, (3) detect QTLs associated with FA proportions and identify candidate genes present within those regions, and (4) estimate the efficiency of genomic selection (GS) compared to pedigree-based BLUP selection using phenotypes predicted by Raman spectroscopy. In this study, we targeted quantification of FA proportions in adipocytes from visceral fat. A reason was that Raman spectroscopy performed on adipocytes is less tedious and expensive than the successive mincing and lyophilisation of flesh reported in previous studies. A second one, was that the procedure proposed could also be usable as highthroughput phenotyping technology by biopsy in a non-destructive manner on live candidates as Raman spectroscopy required a limited size of sample (< 1 g). The methodology of the study encompassing gaz chromatography, Raman spectroscopy, MRI, microwaves and genotyping for calibration step and commercially selected fish was illustrated in Fig. 1.

## Results

### Basic characteristics of fatty acid composition

**Fig. 2:**
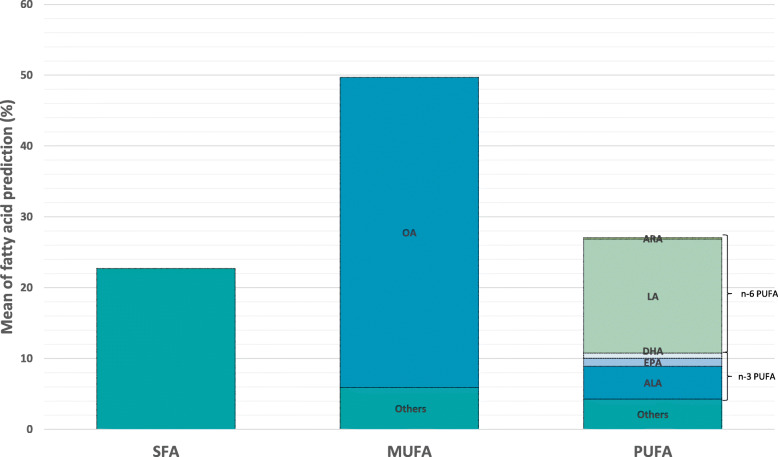
Bar plot showing fatty acid prediction in percentage for SFA, MUFA (OA and others), PUFA (n-3 PUFA (ALA; EPA; DHA); n-6 PUFA (LA; ARA) and others) in rainbow trout

Descriptive statistics of the fish fatty acids (FAs) proportions predicted by Raman spectroscopy in this study are presented in Table 1 and Fig. 2.

**Table 1:** Summary statistics for fatty acid traits in rainbow trout

Name	Trait	N	Mean	SD	Min	Max	CV (%)	R^2^ [50]
SFA	Saturated fatty acids	1382	22.69	1.06	19.2	27.14	4.67	0.42
MUFA	Monounsaturated fatty acids	1382	49.68	0.9	44.33	53.46	1.8	0.75
PUFA	Polyunsaturated fatty acids	1382	27.05	1.37	22	31.29	5.05	0.79
n-3 PUFA	Omega-3 fatty acids	1382	8.87	1.18	4.65	12.39	13.3	0.66
n-6 PUFA	Omega-6 fatty acids	1382	17.19	0.59	14.97	20.06	3.44	0.83
OA	Oleic acid (C18:1)	1382	43.76	1.51	32.99	49.63	3.45	0.85
LA	Linoleic acid (C18:2 n-6)	1382	16.08	0.68	13.05	20.24	4.25	0.84
ALA	Alpha-linolenic acid (C18:3 n-3)	1382	4.62	1.08	0.75	10	23.45	0.82
ARA	Arachidonic acid (C20:4 n-6)	1382	0.21	0.03	0.04	0.31	14.66	0.61
EPA	Eicosapentaenoic acid (C20:5 n-3)	1382	1.11	0.16	0.57	1.86	14.37	0.76
DHA	Docosahexaenoic acid (C22:6 n-3)	1382	0.75	0.12	0.47	1.2	15.63	0.81
EPA + DHA	Sum EPA + DHA	1382	1.97	0.61	0.03	5.04	31.06	0.82

(*N* number of individuals, *SD* Standard deviation, *Min* Minimum value, *max* maximum value, *CV* Coefficient of variation (SD/mean*100), value of R^2^ from the ridge regression methods for calibration from Prado et al. 2021 [50])

In this study, the most abundant individual fatty acid was oleic acid (C18:1, 43.76%), which represented about 90% of all monounsaturated fatty acids (MUFAs) present in visceral fat. Saturated fatty acids (SFAs) made up 22.69% of total FA content, while polyunsaturated fatty acids (PUFAs) represented 27.05%. This latter group was composed of approximately 33% n-3 PUFAs (sum of omega-3 fatty acids) and 64% n-6 PUFAs (sum of omega-6 fatty acids). Of the individual n-6 PUFAs, the most abundant was linoleic acid (LA, 16.08%), which represented ca. 60% of all PUFAs and about 93% of n-6 PUFAs. Of the individual n-3 PUFAs, alpha-linolenic acid (ALA) had the highest concentration, accounting for 50% of n-3 PUFAs and about 17% of total PUFA content. DHA was the second most abundant n-3 PUFA, representing ca. 22% of n-3 PUFAs and 7% of total PUFAs. Taken together, the sum of EPA + DHA corresponded to 35% of n-3 PUFAs and 11% of PUFAs as a whole. Overall, the FA composition of fish was similar to that of the feed (Additional file 1).

Among individuals, we observed relatively little variability in the estimated proportions of different types of FAs (coefficient of variation (CV) ranging from 2 to 5% for SFAs, MUFAs, and PUFAs). Instead, much more variation was detected among individuals in the estimated proportions of individual FAs (CV ranging from 3 to 31%), with the largest CV found for DHA. In general, proportions of n-3 PUFAs were more variable among fish than those of n-6 PUFAs (CVs of 13% and ca. 3%, respectively).

### Heritability estimates and correlations among FAs

**Fig. 3:**
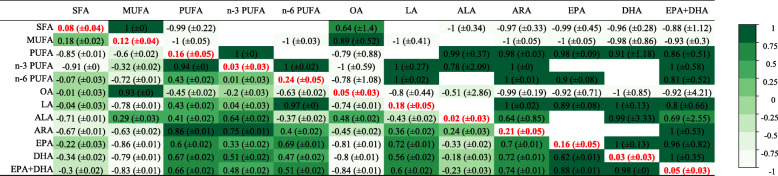
Genetic parameters of fatty acid traits in rainbow trout. Heritabilities (± standard error) in bold red on the diagonal, genetic correlations (± standard error) in the upper triangle, phenotypic correlations in the lower triangle

Figure 3 depicts heritability (*h*^2^) estimates based on pedigree information for individual FAs and FA groups, as well as phenotypic correlations (r_p_) among the 12 traits.

In general, heritability estimates were low and varied from 0.02 ± 0.03 to 0.24 ± 0.05. With respect to FA groups, higher heritability was estimated for PUFAs (*h*^2^ = 0.16 ± 0.05) compared to SFAs (*h*^2^ = 0.08 ± 0.04) and MUFAs (*h*^2^ = 0.12 ± 0.04). Of the two types of PUFAs, n-6 PUFAs had the higher heritability (*h*^2^ = 0.24 ± 0.05). For individual fatty acids, the highest heritabilities were estimated for arachidonic acid (ARA), LA, and EPA (0.21 ± 0.05, 0.18 ± 0.05, and 0.16 ± 0.05, respectively).

Our analysis of phenotypic correlations among traits revealed that, as a group, values for PUFAs as a whole were more strongly correlated to those of n-3 PUFAs than of n-6 PUFAs. Concentrations of ARA, an individual n-6 PUFA, were highly correlated to those of the larger PUFA group, n-3 PUFAs, and the individual FAs EPA, DHA, and sum EPA + DHA. The two types of n-3 LC PUFAs, EPA and DHA, were highly correlated with each other. EPA and sum EPA + DHA were more strongly correlated to n-6 PUFAs, LA, and ARA (r_p_ > 0.5) than to n-3 PUFAs and ALA (r_p_ < 0.5).

When we examined the genetic correlations (r_g_) among FAs, the results were similar to the phenotypic correlations. From a group perspective, SFAs were highly positively genetically correlated to MUFAs (r_g_ = 1 ± 0) and highly negatively genetically correlated to PUFAs (− 0.99 ± 0.22). EPA was highly genetically correlated to LA (0.89 ± 0.08). DHA and the sum of EPA + DHA were positively correlated to LA (r_g_ = 1 ± 0.13, 0.80 ± 0.66, respectively), while the sum of EPA + DHA was also highly correlated to ARA (r_g_ = 1 ± 0.53). However, for the majority of FAs the standard errors of the genetic correlations were high (up to ±2.86 for ALA) and some parameters did not converge, thus some results would need confirmation.

### Correlations between fatty acid proportions and other traits

**Fig. 4:**
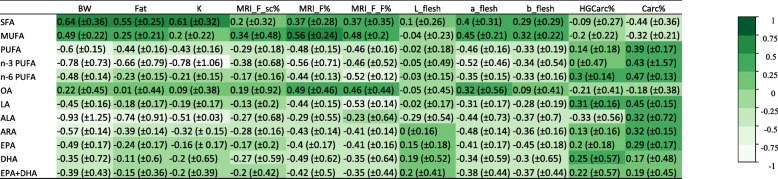
Estimates of genetic correlations between proportions of fatty acids and production or quality traits

Genetic correlations between production traits and fatty acids are presented in Fig. 4 and phenotypic correlations can be found in Additional file 2.

The growth parameters BW and K and fat content parameters Fat, MRI_F%, MRI_F_sc%, and MRI_F_F% were moderately and positively genetically correlated with SFAs and MUFAs (0.2 ± 0.32 to 0.64 ± 0.36). Instead, generally speaking those same production traits tended to be negatively correlated with all PUFA groups and individual PUFAs (− 0.11 ± 0.6 to − 0.93 ± 1.25). BW was highly positively correlated with SFAs and MUFAs (0.64 ± 0.36 and 0.49 ± 0.22, respectively) and highly negatively correlated with PUFAs, n-3 PUFAs, and n-6 PUFAs (− 0.6 ± 0.15, − 0.78 ± 0.73, and − 0.48 ± 0.14, respectively). Fat percentage correlated positively with SFAs and MUFAs (0.55 ± 0.25 and 0.25 ± 0.21), and negatively with PUFAs as a group (− 0.44 ± 0.16) as well as all individual PUFAs (− 0.11 ± 0.6 to − 0.74 ± 0.91).

PUFAs, n-3 PUFAs, and n-6 PUFAs were positively genetically correlated with yield traits (HGCarc% and Carc%), with the highest positive correlation found between Carc% and n-6 PUFAs (0.47 ± 0.13). These yield traits tended to be negatively correlated with SFAs and MUFAs, with the highest negative correlation found between Carc% and SFAs (− 0.44 ± 0.36).

DHA and the sum of EPA + DHA showed a moderate, positive correlation with HGCarc% and Carc% (0.17 ± 0.48 to 0.25 ± 0.57).

We must reiterate here that the standard errors of the genetic correlations were generally quite high; estimates of genetic correlations were thus relatively imprecise.

### QTL detection and identification of candidate genes

**Fig. 5:**
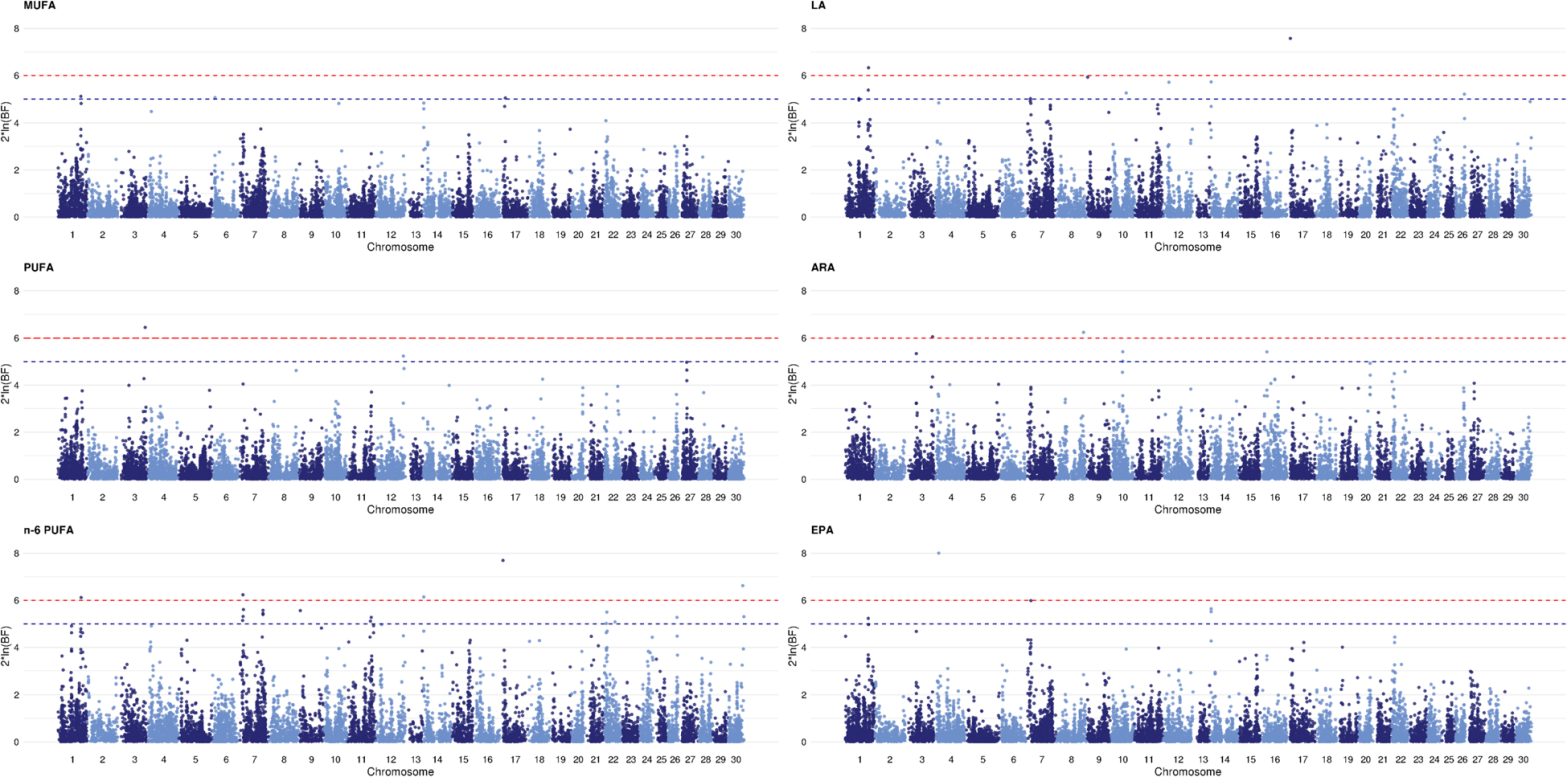
Manhattan plot of QTLs detected using a Bayesian GWAS for fatty acid traits. SNPs are plotted on the x-axis according to their position on each chromosome. The y-axis represents twice the natural logarithm of the Bayes factor associated with each trait. Red and blue dashed horizontal lines correspond to the threshold value of logBF ≥6.0 for definitive evidence of a QTL and the threshold value of logBF ≥5.0 for a putative QTL, respectively

GWAS analyses were performed for the 12 individual FAs or FA groups using approximately 30,000 SNPs. We identified 10 QTLs with evidence effects on the fatty acids proportions as well as 13 putative QTLs with only putative evidence (Fig. 5 and Table 2). Table 2 presents all regions detected for each FA trait and the percentage of total additive genetic variation explained by each. We will first describe the regions for which we detected strong evidence of an effect, then the regions associated with more than one FA, and finally the regions that explained more than 1% of the total additive variance of a trait. Regions associated with only one FA and that accounted for less than 1% of genetic variance are included in Table 2 but will not be described here. The candidate genes identified within the different QTL regions are detailed in Additional file 3.

**Table 2:** Summary statistics for GWAS for fatty acid traits in rainbow trout using BayesCπ method

Trait	Chr.	Peak SNP	SNP peak position (Mb)	2*ln (BF)	MAF	QTLstart position (Mb)	QTLend position (Mb)	% of variance explained by QTL
MUFA	1	AX-89965885	63.63	5.11	0.35	63.60	64.29	0.59
PUFA	3	AX-89945218	75.82	6.45	0.33	75.82	75.82	0.36
PUFA	27	AX-89938879	13.38	4.97	0.16	13.38	14.14	0.64
n-6 PUFA	1	AX-89927799	64.29	6.12	0.33	63.06	64.29	0.97
n-6 PUFA	7	AX-89960795	8.34	6.23	0.29	6.49	10.19	1.67
n-6 PUFA	7	AX-89924956	63.78	5.57	0.37	63.78	65.02	1.29
n-6 PUFA	14	AX-89920031	2.73	6.14	0.31	2.68	2.73	0.42
n-6 PUFA	17	AX-89959128	7.55	7.69	0.37	7.55	7.55	0.61
n-6 PUFA	22	AX-89961755	6.74	5.50	0.45	4.94	8.31	1.6
n-6 PUFA	26	AX-89955255	24.05	5.27	0.43	24.05	25.30	0.56
LA	1	AX-89926288	37.80	5.02	0.29	37.72	37.80	0.62
LA	1	AX-89927799	64.29	6.33	0.33	63.06	64.29	1.08
LA	7	AX-89939304	8.76	5.01	0.25	7.53	10.19	0.86
LA	14	AX-89920031	2.73	5.72	0.31	2.57	2.73	0.45
LA	17	AX-89959128	7.55	7.57	0.37	7.55	7.55	0.64
LA	26	AX-89955255	24.05	5.2	0.43	24.05	24.51	0.19
ARA	3	AX-89945218	75.82	6.06	0.33	75.82	75.96	0.41
ARA	8	AX-89929403	75.66	6.24	0.47	75.66	75.66	0.36
ARA	10	AX-89926441	32.18	5.42	0.28	31.28	32.30	0.94
EPA	1	AX-89965885	63.63	5.23	0.35	63.06	64.29	0.86
EPA	4	AX-89922253	7.94	8.00	0.35	7.94	7.94	0.85
EPA	7	AX-89971360	9.88	5.98	0.37	7.54	10.19	1.02
EPA	14	AX-89920031	2.73	5.64	0.31	2.68	2.73	0.59

*Chr* Chromosome, 2*ln (BF) Twice the natural logarithm of the Bayes Factor, *MAF* Minor allele frequency. The percentage of genetic variance explained by a QTL was calculated as the sum of the variance explained by all SNPs included in the credibility interval of the QTL

Within a single region on Omy7, we identified with evidence effect a QTL for n-6 PUFAs and putative QTLs for LA and EPA. This region explained the highest percentage of genetic variance for any of the traits investigated—1.67, 0.86, and 1.02%, respectively—and also had the largest credibility interval, between 2653 kb and 3705 kb wide. The peak SNPs for n-6 PUFAs, LA, and EPA were located, respectively, at 8.34 Mb (coiled coil domain-containing protein 122-like), 8.76 Mb (intergenic region between *tnika* (traf2 and NCK-interacting protein kinase-like) and *slc7a14b* (probable cationic amino acid transporter) genes), and 9.88 Mb (intergenic region between *ino1a* (inositol-3-phosphate synthase 1-A-like) and *gnao1a* (guanine nucleotide-binding protein subunit alpha-11-like) genes) respectively.

Between 63.06 Mb and 64.29 Mb on Omy 1, we detected QTLs for n-6 PUFAs and LA, and putative QTLs for MUFAs and EPA. Depending on the trait, this region explained between 0.59 and 1.08% of the genetic variance and had a credibility interval that was between 688 kb and 1232 kb wide. The peak SNP for n-6 PUFAs and LA, at 64.29 Mb, was located in the intergenic region between *tlx1* (T-cell leukaemia homeobox protein 1 isoform X1) and *lbx1* (transcription factor LBX1-like). The peak SNP for MUFAs and EPA, at 63.63 Mb, was located in the PH and SEC7 domain-containing protein 1-like isoform region.

A region on Omy3 (located between 75.82 Mb and 75.96 Mb) contained QTLs for PUFAs and ARA, but these explained less than 0.5% of the genetic variance in each trait. The peak SNP for these QTLs was located in the intergenic region between *tmtops2a* (vertebrate ancient opsin-like) and *jam2b* (junctional adhesion molecule B-like isoform X1).

A region on Omy14 (located between 2.57 and 2.73 Mb) contained a QTL for n-6 PUFAs and putative QTLs for LA and EPA. This region explained a low percentage of the genetic variance in these traits—0.42, 0.45, and 0.59%, respectively—and had credibility intervals that spanned from 50 to 165 kb. The peak SNP for this region was located at 2.73 Mb, in the intergenic region between *rims2b* (regulating synaptic membrane exocytosis protein 2-like) and *rims2a* (regulating synaptic membrane exocytosis protein 2-like isoform X1).

On Omy26, one region was identified as a putative QTL for both n-6 PUFAs and LA. This region explained 0.56 and 0.19% of genetic variance, respectively, and was located between 24.05 Mb and 25.30 Mb. The peak SNP for this QTL was located at 24.05 Mb, in the intergenic region between *rbtn1* (rhombotin-1) and *rergl* (ras-related and estrogen-regulated growth inhibitor-like protein).

Two other putative QTLs were detected that each explained more than 1% of the genetic variance associated with PUFAs as a group. The first was located on Omy7, had a credibility interval of 1239 kb, and explained 1.29% of the genetic variance for this trait. The peak SNP for this QTL was located at 63.78 Mb, in the *znf1007* (zinc finger protein 595-like) gene. The second putative QTL was located on Omy22 between 4.94 and 8.31 Mb, and explained 1.6% of genetic variance. The peak SNP for this QTL was located in the *lmo7* (LIM domain only protein 7) gene at 6.74 Mb.

No significant QTL was found for SFAs, n-3 PUFAs, OA, ALA, DHA, DPA, and EPA + DHA, which suggests that the underlying genetic architecture for these traits is very polygenic and affected by multiple loci with small effects in this population of rainbow trout.

### Genomic selection

The accuracies of estimated breeding values (EBVs) and genomic estimated breeding values (GEBVs) are shown in Table 3 for all FAs; data on the efficiency of genomic selection, along with the inflation coefficients, can be found in Table 3.

**Table 3:** Mean and standard deviation (in brackets) over 40 replicates of the selection accuracy (Acc) and inflation coefficient (b) of EBVs and GEBVs for fatty acid traits

	BLUP		GBLUP	
	Acc	b	Acc	b
SFA	0.37 (0.13)	1.07 (0.45)	0.45 (0.14)	1.03 (0.39)
MUFA	0.38 (0.11)	0.99 (0.34)	0.58 (0.11)	1.11 (0.28)
PUFA	0.43 (0.12)	1 (0.37)	0.56 (0.11)	1.01 (0.27)
n-3 PUFA	0.31 (0.24)	1.27 (1.03)	0.36 (0.23)	1.1 (0.72)
n-6 PUFA	0.51 (0.08)	1.03 (0.2)	0.7 (0.07)	1.08 (0.16)
OA	0.26 (0.18)	0.95 (0.67)	0.5 (0.19)	1.32 (0.58)
LA	0.46 (0.09)	1.02 (0.25)	0.67 (0.09)	1.12 (0.18)
ALA	0.3 (0.3)	1.8 (1.85)	0.34 (0.25)	1.42 (1.06)
ARA	0.46 (0.1)	1 (0.28)	0.51 (0.09)	0.91 (0.21)
EPA	0.43 (0.1)	0.99 (0.27)	0.53 (0.09)	0.98 (0.22)
DHA	0.21 (0.23)	1 (1.09)	0.47 (0.23)	1.53 (0.83)
EPA + DHA	0.27 (0.17)	0.96 (0.64)	0.46 (0.17)	1.21 (0.49)

Regardless of the trait in question, all inflation coefficients were statistically indistinguishable from 1 (see Table 3) and nearly identical between pedigree-based (BLUP) and genomic (GBLUP) approaches.

GEBVs were more accurate than EBVs for all of the predicted traits. The accuracy of genomic selection was approximately 45% higher than that of pedigree-based selection for all FAs. The mean accuracy of GEBVs varied from 0.34 for ALA to 0.70 for n-6 PUFAs, whereas the corresponding accuracies of EBVs were 0.30 and 0.51, respectively. The highest increase in accuracy for a GEBV with respect to the corresponding EBV was obtained for DHA (+ 119.8%), while the lowest was obtained for ARA (+ 11.8%).

## Discussion

In this study, we estimated the genetic parameters of FA composition predicted by Raman spectroscopy and the correlations between FA traits of visceral adipocytes and those associated with production and quality traits (body weight, carcass and filet yields, and lipid deposition) in a commercial population of rainbow trout. We identified quantitative trait loci and evaluated the potential for genomic selection aimed at increasing the abundance of long-chain omega-3 polyunsaturated fatty acids in these fish.

### Raman phenotyping and sampling of visceral adipose tissue

In the majority of previous studies on aquatic species, genetic variability in FA composition has been evaluated using chemical analyses and gas chromatography [19, 20, 31, 33, 52, 53]. Due to the high cost of these analyses, such studies have typically relied on relatively small datasets, which limits the usefulness of this approach for rigorous genetic evaluation and selection. The inclusion of FA traits in breeding programmes would thus depend greatly on the availability of alternative methods for FA analysis. The development of breeding programs could also benefit from alternative non-destructive technologies to predict FA on live candidates. For this reason, the present study investigated the use of Raman spectroscopy, which is less expensive than GC-based analyses and would therefore enable the construction of the larger datasets (thousands of individuals) necessary for study of genetic architecture. As reported previously, this technology requires also a small size of tissue (few μm^3^) that could be frozen. Thus it could be a non-lethal biopsy applied on live candidates, making it affordable in breeding programs.

The accuracy of this technique is determined by calibration equations that are constructed using GC-based FA quantification [Prado E, Eklouh-Molinier C, Enez F, Causeur D, Blay C., Dupont-Nivet M, Labbé L, Petit V, Moreac A, Taupier G, Haffray P, Bugeon J, Corraze G, Nazabal, V. Prediction of fatty acids composition in the rainbow trout Oncorhynchus mykiss by using Raman micro-spectroscopy. Submitted]. For the majority of FAs (MUFAs, PUFAs, n-6 PUFAs, OA, LA, ALA, EPA, DHA, and EPA + DHA), the quality of prediction was high (correlation coefficient (R^2^) ≥ 0.75, as estimated using ridge regression methods). Only three of the FA traits tested (SFAs, ARA, and n-3 PUFAs) demonstrated weaker predictive power, with R^2^ < 0.75 [50]. One potential disadvantage of this technique, however, could be its limit of detection, which is around 1%; some of the values for individual FAs in the current study were close to this percentage. Curiously, though, the predictive power for the SFA group was quite limited (R^2^ = 0.42), even though the proportions calculated for this group were well above the detection threshold (around 22%). Further investigations are currently underway to study these results more thoroughly.

Another potential point of contention could be the use of adipocytes from visceral fat instead of minced fillet or adipocyte cells from myosepta. Major reasons of this choice were detailed in the introduction. Adipocytes are located in various adipose tissues (mostly visceral, subcutaneous, liver, red and white muscles, brain, pancreas, mandible, cranium, and tail fin); they play an important role in the long-term storage of FAs as triglycerides (lipogenesis) and their subsequent release (lipolysis) in the blood for use in cell growth, endocrine regulation, reproduction, or as energy sources after β-oxidation [54, 55]. The processes of adipocyte multiplication (hyperplasia) or growth in size (hypertrophy), as well as lipolysis, are regulated by a variety of enzymes, transcription factors, and hormones that also interact with the enzymes fatty acyl elongase (Elovl) and desaturase (Fad) to yield the final edible FA composition of the muscle. Genetic variability in the FA composition of the fillet results then from all the previous physiological process acting in interaction with important environmental factors such as swimming activity, water temperature, feed composition, or feeding practices. At this time, there is no published information about potential differences in FA composition between adipocytes in different locations in the fillet. In targeting visceral adipocyte composition, we aimed to evaluate genetic differences in lipogenesis- and lipolysis-associated processes while minimising, as much as possible, local interaction with other cell types or tissues. This may limit the applicability of our results to this type of tissue. The proportion of polar lipids is higher in muscle (around 25%) than in adipose tissue (5–10%). The n-3 long-chain PUFAs are mostly associated with sn-2 positions of phospholipids, so we can reasonably hypothesise that the relative proportion of n-3 LC-PUFA (in % of total FA) would be higher in muscle than in adipose tissue and then can be better predicted by Raman spectroscopy due to their higher abundance (in proportion). Our study provides a starting point—and a new technical methodology—for the characterisation of potential genetic variation of FA composition and will need further investigation to compare muscle and visceral FA composition.

### Heritabilities for fatty acid composition and correlations

To our knowledge, this study is the first to report pedigree heritability estimates for all FA groups and individual FAs in rainbow trout. An earlier study in the same species estimated heritability for EPA (0.61 ± 0.17) and DHA (0.77 ± 0.11), but with a limited study population (220 fish from 44 families) [52]. Another study on Atlantic salmon reported a similarly high estimate for the n-3 PUFA group (0.77 ± 0.14) [19]. These estimates are very different than those we obtained for EPA (0.17 ± 0.05) and DHA (0.03 ± 0.03), probably due to differences in population structure, phenotyping method, and/or in the age and diet of the studied fish. In our case, the contrast between the limited proportions of PUFAs (15% of total FAs), EPA (1–2.3% of total FAs), and DHA (around 1% of total FAs) and the much-larger proportions of MUFAs (47–56% of total FAs) and SFAs (14–27% of total fat) may have hindered our ability to accurately estimate the underlying genetic variation. However, in general, our results are consistent with those documented from many other aquatic species. For example, in a study of Atlantic salmon whose diet contained fish meal and fish oil, and thus higher PUFA concentrations than in the present study, Horn et al. reported heritabilities that were similar to ours, in the range of 0.09 ± 0.06 to 0.26 ± 0.08 for individual n-3 PUFAs [20]. Although there were large differences between the two studies in the FA composition of the feed (EPA + DHA made up 3% of total dietary fat in the trout feed used here, compared with 17% in their salmon feed; trout feed was 25–30% fat versus 36% for salmon), this does not seem to have influenced the heritability estimates. For the shrimp *Litopenaeus vannemei*, published heritability estimates were even lower, ranging from 0 to 0.19 ± 0.07 for all fatty acids, 0.07 ± 0.05 for EPA, and 0.12 ± 0.06 for DHA [53]. In tilapia, heritability estimates ranged from 0 to 0.39 ± 0.11, with 0.10 ± 0.08 for EPA and 0.004 ± 0.07 for DHA [31]; in common carp, estimated heritabilities ranged from 0.03 ± 0.10 to 0.37 ± 0.22, with only three estimates different from zero: PUFAs (0.29 ± 0.17), n-3 PUFAs (0.37 ± 0.22), and EPA (0.34 ± 0.20) [33]. Thus, our estimates for the heritability of FA groups or individual FAs are in the same range as previous results obtained from other aquaculture species. Although these estimates were globally low, they do point to a genetic component in rainbow trout for FA composition and abundance, particularly for n-6 PUFAs, which could be exploited through genetic selection. The higher heritabilities we observed for n-6 PUFAs, both as a group and for individual FAs, were most likely due to the improved prediction accuracy for these FAs compared to SFAs, MUFAs, or n-3 PUFAs (both as a group or as individuals). It could be that SFAs or MUFAs have low heritabilities because they are mainly modulated by the diet, which in this study contained very high proportions of these FAs. Additional information from multiple species may help us to better understand the genetic determinism in the FA transformation cascade and the respective influences of the elongase and desaturase enzymes in different contexts.

Strong genetic correlations, whether positive or negative, between different fatty acids is favourable to select the trait with the highest heritability. The genetic correlations estimated in the present study among FAs and traits associated with body weight, yield, and quality enable us to make predictions about changes that may occur as a result of selection on a given trait. Generally, our results indicated that improvement in one FA group is likely to cause unfavourable changes in other groups. In our study, the negative genetic correlations between PUFAs and SFAs/MUFAs demonstrate the trade-offs between groups. Generally, the genetic associations were synergic within FA groups but antagonistic between groups, which arises as a result of constraints inherent in the underlying biosynthesis pathways of these FAs. Additionally, even though our dataset of 1382 fish was larger than those used in previous studies, only some of the bivariate models for individual fatty acids converged (due to very high correlations between those pairs of traits) and all genetic correlations had large standard errors. Thus, we based our interpretation of the results on group traits rather than on individual estimates.

Another important contribution of this study is our characterisation of the genetic correlations between FAs and traits related to body weight, yield, and quality. In rainbow trout, and in fish in general, the main selection objectives are faster growth, increased yields, and disease resistance. Given the genetic correlations estimated here, genetic selection for increased BW or fat content would be expected to increase the proportions of SFAs and MUFAs and decrease the proportion of PUFAs; such a reduction in the abundance of healthful n-3 PUFAs could diminish the nutritional quality of flesh. Similar results have been observed in studies of common carp [33], Atlantic salmon [19], and tilapia [31], in which unfavourable genetic correlations were reported between performance traits and EPA or n-3 PUFAs. The fact that the same tendency we observed in visceral adipocytes has been reported in other studies based on fillets indicates that adipocytes probably exhibit similar relative variations in FA composition regardless of their origin.

One explanation of why faster growth or yield improvement could drive differential lipid composition may also be linked with nutrition. It is well known that the FA composition of diets strongly affects the composition of lipids in fish [16, 56–58]. In a 2018 study, trout that were selected for faster growth tended to have a higher feed intake; the fish then stored unutilised energy as MUFAs, which shifted the balance of FAs in flesh away from PUFAs [33]. Here, we identified a moderate positive genetic correlation between fillet yield (predicted by HGCarc%) or carcass yield (Carc%) and certain FA traits (DHA and sum EPA + DHA). This could be linked to the fact that, compared to oleic acid (18:1 n-9), EPA and DHA lower the accumulation of triglycerides in adipocytes, thus limiting the development of adipose tissue in fish [54, 59]. Carcass yield is inversely correlated to visceral yield (with increased visceral tissue, carcass yield declines), so it is likely that as the amount of visceral tissue increases, we would see a corresponding decline in EPA and DHA content. Our results give support to this hypothesis on the link between EPA and DHA and oleic acid storage and the interest to limit fat deposition in the muscle and/or the visceral fat tissue in selecting leaner fish with higher fillet yield. Thus selecting for increasing carcass yield should be a good strategy which should preserve or increase EPA and DHA content, therefore maintaining a favourable balance of FAs and the accompanying nutritional benefits for humans [55]. Such an approach might be preferable to one based only on growth, which is negatively correlated to n-3 LC PUFA content. However, selecting for both carcass yield and EPA/DHA content would reinforce the impacts on FA composition.

### New QTLs and candidate genes for fatty acid composition

All previous studies on the genetic basis of FA composition in aquaculture species have described a polygenic architecture, with a few QTLs responsible for moderate levels of genetic variation in FAs [28, 32, 33, 44, 60].

Our study identified QTLs on Omy7 that were associated with n-6 PUFAs, LA, and EPA; contained within this region was the gene *onmy-cd8a* (T-cell surface glycoprotein CD8 alpha precursor), which is indirectly related to FA activity. In Atlantic salmon, a gene in the same family (*LOC106581970*, T-cell surface glycoprotein CD3 zeta chain-like, on chromosome 21) was also identified as a potential candidate gene with links to EPA and DHA [28]. It has been hypothesised that EPA and DHA are able to modulate T-cell activation to exert anti-inflammatory influences [61–63]. Within this same region on Omy7, we also identified *mrps9* (28S ribosomal protein S9, mitochondrial isoform) near the peak SNP. Members of the MRPS family are involved in the synthesis of protein inside mitochondria [64], with one of their roles being the induction of apoptosis by SFAs in several cell types [65, 66]. Another member of this family, *MRPS30* (mitochondrial ribosomal protein S30), was highlighted by a GWAS analysis on FA composition in sheep [64]; this gene was associated with myristic acid (C14:0) content and the ratio of n-6 to n-3 PUFAs.

On Omy1, we detected QTLs associated with n-6 PUFAs, MUFAs, LA, and EPA; close to the peak SNP in this region, we identified three interesting genes with indirect links to FAs: *apmap*, *accs1*, and *abhd12*. *Apmap* (adipocyte plasma membrane associated protein) has arylesterase and strictosidine synthase activity and may play a role in adipocyte differentiation [67], while *acss1* (acetyle coenzyme A synthetase 2-like) catalyses the synthesis of acetyl-CoA from short-chain FAs [66]. *Abhd12* (Lysophospholipase ABHD12) is known to be involved in immune and neurological processes and plays a role in the regulation of lysophospatidylserine pathways and related with very-long chain lipids [68–70].

In the ARA-associated QTL on Omy10, the gene *acsl3a* (long-chain fatty acid CoA ligase 3-like) was present close to the peak SNP [71]. This gene belongs to the same family as Acyl-coenzyme A (CoA) synthetase 1 (*acsl1*), a well-studied obesogenic gene involved in FA metabolism that is associated with high caloric food intake in mice and humans [72]. In general, members of this family appear to play similar roles: *acsl5* has been implicated in lipid biosynthesis and FA degradation [73], and *acsl1* and *acsl5* were linked with ARA and ARA/ALA, respectively, in a study of common carp [42]. Another gene of interest present in this region was *mogat3b* (monoacylglycerol O-acyltransferase 3b), which is predicted to have diacylglycerol O-transferase activity; it also plays a role in triglyceride biosynthesis and may be involved in the absorption of dietary fat [74–76]. An important paralog of this gene is *dgat2* (diacylglycerol o-transferase 2), which has been implicated in the catalysis of the final stage of triacylglycerol biosynthesis [77] and linked to adipogenesis [78]. This gene was also highlighted in a study on sheep, in a QTL associated with SFAs, C18:0, C16:1, and MUFAs [79].

In a QTL associated with PUFAs on Omy12, we found the gene *LOC110538527*, which encodes the butyrophilin subfamily 1 member A1 protein. Butyrophilin is the main protein associated with milk fat droplets and milk quality in cattle [80], and several members of this family were highlighted in a GWAS analysis of FA composition in beef cattle [34].

The candidate gene *TBC1D4* was found close to the peak SNP in a QTL on Omy22 associated with n-6 PUFAs. Members of the TBC1 domain family have been associated with insulin and FA composition in previous studies of humans and mice, and warrant further investigation [81–83]. In pigs, this gene family, and more specifically, *TBC1D1*, was also reported to have putative effects on three FA ratios (C16:1 n-7/C16:0, C18:1 n-9/C16:1 n-7, PUFA/MUFA), on PUFAs as a whole, as well as on n-3 and n-6 PUFAs [84].

Some previous studies of the genetic basis of FA content have identified candidate genes linked to FA metabolism, such as the *elovl* or *fad* genes in pigs [85] or *elovl2* (involved in the conversion of DPA to DHA) in Atlantic salmon [28]. In our study, we did not detect any strong candidate genes that were directly involved in the bioconversion of PUFAs, n-3 PUFAs, EPA, or DHA in rainbow trout. However, we did note several candidate genes with links to EPA-, DHA-, or PUFA-related traits. Other candidate genes highlighted by our analysis included *sdhaf4* (succinate dehydrogenase assembly factor 4, mitochondrial-like), free fatty acid receptor 2-like, *atp11a* (phospholipid-transporting ATPase IH isoform1), *plpp2a* (phospholipid phosphatase 2-like), isocitrate dehydrogenase, *pemt* (phosphatidylethanolamine N-methyltransferase), *inpp4ab* (Inositol polyphosphate-4-phosphatase type I Ab), *mtmr2* (Myotubularin-related protein 2), *ebpl* (EBP-like), *dhdds* (alkyl transferase), and *sstr5* (Somatostatin receptor 5). Several factors could explain the lack of association with n-3 PUFA or n-6 PUFA bioconversion pathway genes. One explanation could be that the SNPs on the array are not located in gene regions that influence lipid metabolism, or that any such genes located near the SNPs remain uncharacterised. It could also be that the diversity of EPA- and DHA-pathway genes is low, which would reduce our ability to detect QTLs. Finally, GWAS efficiency is influenced by the accuracy of phenotypic recording. It is possible that our decision to predict FA composition using Raman spectroscopy, which is less accurate than gas chromatography, might have influenced the result.

### Genomic selection for fatty acids

Unlike pedigree-based BLUP selection, in which candidates from the same family have the same EBV, genomic selection (GS) is able to differentiate among candidates within a family and may thus yield improved estimates of breeding values and better selection efficiency. In particular, GS is more efficient than pedigree-based selection for traits which cannot be measured directly on selection candidates and for traits with limited heritability, such as FA proportions. Furthermore, GS has the additional advantage of requiring fewer phenotypes, since only the reference population must be phenotyped. This is especially beneficial for traits that are complex and expensive to measure, such as FA composition. In our study, GS for FA traits using a reference population of 1100 individuals was estimated to improve accuracy by 12 to 120% compared to BLUP selection. In the literature to date, only one study has compared the accuracy of selection for FA content in fish (in this case, Atlantic salmon) between pedigree- and genomic-based methods [86]. Those authors reported low to moderate GS accuracies (0.27 to 0.61), which are similar to the estimates we found here (0.34 to 0.70). For DHA, the pedigree and genomic accuracies were 0.33 and 0.41, respectively (a 26% gain in accuracy with GS, compared with 120% in our study), while for EPA, these values were 0.37 and 0.32, respectively (a higher accuracy for pedigree prediction; − 14% change in accuracy for GS compared to the 23% gain estimated in our study). In studies of cattle, the accuracy of GS was less than 0.40 for the majority of FAs [34, 36] and the reliability of genomic prediction for milk FA composition from three Holstein populations was also less than 0.40 [87]; despite this, though, genomic prediction was always superior to pedigree prediction. In our study, the two lowest values of genomic accuracy were obtained for n-3 PUFAs and ALA, which were also the two FAs with the lowest heritabilities. Compared to traditional pedigree-based selection, GS seems to demonstrate particular potential for the healthy, desirable FAs EPA, DHA, and EPA + DHA (gains of + 23%, + 120%, and + 70%, respectively).

Genotyping and data collection are costly, and the relative advantage of using SNP data in selection ultimately depends on how these costs are offset by the value of the improvement in traits of interest. The higher genetic gains enabled by GS with respect to pedigree selection may partially cover the extra costs of genotyping [88]. Because of this, it may be feasible to incorporate GS for FA traits in breeding programmes in order to select fish with a superior genetic basis for FA composition. The major advantage of GS is the ability to differentiate among candidates within a family, which here resulted in a gain in accuracy between 12% and 120%; this advantage is particularly pronounced for traits that cannot be measured directly on a selection candidate. Furthermore, the cost of implementation of GS could be reduced by optimising the density of the SNP panel in use. Several studies have evaluated the accuracy of predictions made using more cost-effective lower-density SNP chips, and, for the traits examined, the use of 500-SNP panels still provided predictive accuracy that was higher than that of BLUP [89, 90].

## Conclusions

In summary, this work provides new insights into the genetics of fatty acid traits in rainbow trout. Our results reveal that fatty acid proportions are very polygenic traits, but, under the conditions investigated here, most appear to be moderately heritable, with the highest heritability observed for n-6 PUFAs (0.24). We detected several genomic regions that explained up to 2% of the genetic variance in proportions of MUFAs, PUFAs, n-6 PUFAs, LA, ARA, and EPA. When we investigated these regions, we identified several genes (*mrps9*, *mogat3b*, *TBC1D4, acsl3*, *onmy-cd8a,* butyrophiulin family, *apmap*, *acss1*, and *abhd12*) that can be indirectly implicated in fatty acid metabolism. These genes represent good candidates for further functional validation to decipher the biological mechanisms underlying variation in fatty acid traits in rainbow trout. The work also provides new information allowing to propose that selection on carcass or fillet yields should improve n-3 LC PUFA composition of the fillet, but a combined approach based on both yield and FA composition in the fillet should further increase the efficiency of selection. Finally, our analyses indicate that, with a reference population of about 1100 individuals, the implementation of genomic selection in a breeding programme for fatty acid traits would enable a gain in accuracy of 12–120% compared to standard pedigree-based selection. These results suggest that genomic evaluation is a feasible strategy for selecting trout with superior genetic merit for traits related to production, quality, and fatty acid composition.

## Methods

### Fish production and trait recording

The fish used in this study were derived from a commercially selected line from the Sources de l’Avance breeding company, a subsidiary of Aqualande Group (Pissos, France). The line has been previously selected for 9 generations with a multi-trait selection combining mass selection on growth and carcass yield assisted by ultrasound and morphometry and sib selection on carcass yield and fillet yield [91].

Through 10 factorial crosses, 84 dams were crossed with 99 neomales (sex-reversed females used as sires) on the same day to create 831 families of rainbow trout. A piece of fin was sampled from each parent for DNA extraction and subsequent genomic analysis. Trout were reared under commercial conditions in the “Viviers de la Houtine” growing farm (Belin-Beliet, France); further details on rearing can be found in [46]. Fish were fed to satiation using extruded commercial feed: Neo start (17% lipids) and Neo CDC (23% lipids) (Le Gouessant, Lamballe, France) during the first stage, then Extra CDC AQL G25 (25% lipids) (Le Gouessant, Lamballe, France) and Viva Pro 7F NAT29 (30% lipids) (Aqualia, Arue, France) until the end of the experiment. The composition of the feed mimicked that used in commercial operations: the majority of fish meal and fish oil had been substituted with plant-based ingredients, so that PUFA levels were minimal (detailed in Additional file 1). Fish were reared following standard practices, and measurements were performed only on slaughtered fish; there was thus no need to consult an ethics committee. At 469 days post-fertilisation (dpf), fish were individually tagged with RFID transponders and fin samples were collected and preserved in 95% ethanol for DNA extraction and genomic analysis. At the end of the growing period (between 503 and 506 dpf), data were collected from 1410 fish randomly sampled. Fish were humanely killed by a blow to the head and bled by cutting the gills in an ice water bath, in accordance with good animal slaughtering practices. Post-mortem data collection and processing were accomplished as quickly as possible to ensure data accuracy. All measured traits were defined according to the ATOL (Animal Trait Ontology for Livestock) database, available online (https://www.atol-ontology.com/en/). From each fish, the following traits were measured: body weight (BW, ATOL_0000351), body length (BL, ATOL_0001658), head weight (HeadW, ATOL_0001545), headless gutted carcass weight (HGCarcW, ATOL_0002260), and viscera weight (ViscW, ATOL_0002258). These traits were combined to calculate three synthetic traits or processing yields: Fulton coefficient condition, calculated as K = BW(g)∗100/BL^3^ (cm) (ATOL_0001653); headless gutted carcass yield (HGCarc%, ATOL_0002261), an indirect predictor of fillet yield (rg = 0.97 [92];); and gutted carcass yield (Carc%, ATOL_0000548). Total fat content in muscle (Fat, ATOL_0001663) was estimated indirectly using a Fish Torry Fat-meter® positioned on the skin as described in [93]. For each fish, one steak was cut in front of the dorsal fin and photographed using a digital camera (Canon EOS 1000 D 10 M Pixels, adapted with a shooting tent (Literoom Photoflex©) to avoid specular reflection and a copy stand to fix the camera. The steaks were then packed in individual plastic bags and frozen at − 20 °C until magnetic resonance imaging (MRI) analysis. The digital pictures were analysed using a modification of the method described by Marty-Mahe et al. (2003) [94]. First, the image was L*a*b* transformed, then colour image segmentation was performed with Visilog 7.3 for Windows© to quantify areas of the steak (ATOL_0005553). Flesh colour (ATOL_0001017) (including myosepta) was expressed in the L*, a*, b* system—representing luminosity, redness and yellowness, respectively—as recommended by the CIE (CIE 1976) [95].

The process of magnetic resonance image formation is based on a combination of permanent and radio-frequency (RF) magnetic fields [96]. An automatic image analysis scheme can then be used to distinguish flesh from subcutaneous fat tissue. The method for mapping the distribution of fat is detailed in [97]. For each steak, MRI measurements were used to determine total fat content (MRI_F%), subcutaneous fat proportion (MRI_F_SC%), and fat content in flesh (IRM_F_F%). The genetic architecture of all of these traits has already been analysed, and further details on trait measurement can be found in additional Table 4 and Blay et al. (2021) [46]. Data from that study are used here to estimate the phenotypic and genetic correlations of production traits with fatty acids.

### Prediction of fatty acid composition

The FA composition of adipocytes from visceral fat (ATOL_0000074) was predicted by Raman spectroscopy (LabRAM HR800, Horiba Scientific) following the calibration method developed by [Prado E, Eklouh-Molinier C, Enez F, Causeur D, Blay C., Dupont-Nivet M, Labbé L, Petit V, Moreac A, Taupier G, Haffray P, Bugeon J, Corraze G, Nazabal, V. Prediction of fatty acids composition in the rainbow trout Oncorhynchus mykiss by using Raman micro-spectroscopy. Analytical Chimica Acta, submitted], with gas chromatography (Eurofins Analytics, Nantes) as a reference method [98]. The detection limit for gas chromatography was estimated to be 0.05% of total FAs. This is detailed in [Prado E, Eklouh-Molinier C, Enez F, Causeur D, Blay C., Dupont-Nivet M, Labbé L, Petit V, Moreac A, Taupier G, Haffray P, Bugeon J, Corraze G, Nazabal, V. Prediction of fatty acids composition in the rainbow trout Oncorhynchus mykiss by using Raman micro-spectroscopy, submitted] but briefly (see also Fig. 1), the calibration was made from 1) 259 individuals divided into two groups fed with two different diets, one enriched in marine fish oil and fish meal and one substituted with plant ingredients 2) nine additional individuals from the 1410 individuals of this study. Visceral adipose tissues were analysed by gas chromatography and also by Raman spectroscopy for 268 samplesand only with Raman for the rest of the samples (1401).

Visceral fat (2 g), mostly composed of adipocytes, was collected from the “front” lobe of visceral adipose tissue from 1410 commercial trout and preserved in liquid nitrogen. Raman measurements were acquired on 1410 sibs (including the 9 used for the calibration) using a 10x objective and an excitation wavelength of 785 nm. Two spectral ranges were recorded: 550 to 1800 cm^− 1^ and 2610 to 3100 cm^− 1^. The calibration by means of a ridge regression model was used to predict FA composition in the 1410 rainbow trout. Specifically, the proportions of saturated fatty acids (SFAs), mono-unsaturated fatty acids (MUFAs), poly-unsaturated fatty acids (PUFAs), omega-3 (n-3 PUFAs) and omega-6 (n-6 PUFAs) fatty acids, and several individual fatty acids were calculated as a percentage of total FA content. The acquisition parameters, pre-processing treatment, and statistical analyses are described in detail by Prado et al. (2021) [Prado E, Eklouh-Molinier C, Enez F, Causeur D, Blay C., Dupont-Nivet M, Labbé L, Petit V, Moreac A, Taupier G, Haffray P, Bugeon J, Corraze G, Nazabal, V. Prediction of fatty acids composition in the rainbow trout Oncorhynchus mykiss by using Raman micro-spectroscopy, submitted.].

### Genotyping

Fish were genotyped using the 57 K SNP Axiom® Trout Genotyping array from Thermofisher [99] at the INRAE genotyping platform Gentyane (Clermont-Ferrand, France).

SNPs were subjected to several steps of quality-control filtering, as described in D’Ambrosio et al. (2019) [100], in particular to remove SNPs with probe polymorphism and multiple locations on the genome. From the initial set of 57 K SNPs, we retained those with a call rate higher than 0.97, no significant deviation from Hardy-Weinberg equilibrium (*p*-value > 0.0001), and a minor allele frequency (MAF) higher than 0.05 for further analysis. In addition, samples in which less than 90% of SNPs were genotyped were removed from the analysis. Finally, all missing SNP genotypes of the remaining individuals were imputed using FImpute software 2.0 [101]. A total of 29,652 SNPs passed quality-control filtering. Following DNA quality control, our final dataset contained genotypes for 1382 of the 1410 fish that had been phenotyped for FA traits.

### Estimation of genetic parameters

Heritability (*h*2) and phenotypic and genetic correlations (*r*p, and *r*g, respectively) were estimated using the restricted maximum likelihood method (AIREML) and BLUPF90 software [102]. Univariate analyses were performed to estimate the heritability of all traits. Bivariate analyses were performed to estimate genetic correlations between traits, using the following animal model:

$$ {Y}_i=\upmu +{\mathrm{a}}_i+{\mathrm{e}}_i $$

where Y_i_ is the performance of the i^th^ animal, μ is the overall mean of the population, a_i_ is the additive effect of the i^th^ animal, and e_i_ is the residual random error term. The pedigree under consideration contained 17,235 animals over 9 generations. The maternal variance was not significantly different from zero and thus maternal effects were not included in the final models for all traits. All fish were female and reared in the same raceway, so there was no need for a fixed effect to be included in the model.

Heritability estimates were calculated as the ratio of additive genetic variance (Va) divided by the total phenotypic variance (Vp). For the genetic correlation among FAs, data were transformed using a logarithmic function to improve the convergence of estimates.

### GWAS and QTL detection

GWAS was performed using BayesCπ [103], a Bayesian Stochastic Search Variable Selection approach, implemented in BESSiE software (version 1.0) [104].

In the BayesCπ model, only a certain proportion of SNPs (π) are assumed to have a non-zero effect on the phenotype. The marker effects are estimated through an MCMC algorithm that considers a mixture of markers, of which proportion π have effects that follow a normal distribution N (0, σ^2^_a_) and proportion 1 – π have zero effect. The general model used is:

$$ {Y}_i=\mu +\sum \limits_{j=1}^n{\delta}_{jk}{a}_j{g}_{ij}+{\varepsilon}_{ik} $$

with **Y** the phenotype observed for the i^th^ individual, μ the overall mean in the population, n the total number of SNPs in the analysis, a_j_ the additive effect of the reference allele for the j^th^ SNP, g_ij_ the genotype for individual i (coded as 0, 1, or 2), and *ε*_*ik*_ the residual effect for the i^th^ individual in the k^th^ iteration. The vector of residual effects is normally and independently distributed, $\boldsymbol{\varepsilon} \sim N\left(0,\boldsymbol{I}{\sigma}_e^2\right),$ with σ^2^_e_ the residual variance.

A total of 200,000 cycles were used, with a burn-in period of 5000 cycles. Results were saved every 20 cycles. In order to check convergence, the MCMC algorithm was initiated three times with three different chains for the random number generator. Convergence was assessed by visual inspection of plots of the posterior density of genetic and residual variances and by high correlations (r > 0.99) between the genomic estimated breeding values (GEBVs) estimated from the different chains of the MCMC algorithm.

At each cycle k, the decision to include the j^th^ SNP in the model depended on the indicator variable δ_jk_: if δ_jk_ was equal to 1, the effect of the j^th^ SNP was estimated as a_j_, while if δ_jk_ was equal to 0, no effect was estimated. This indicator variable was sampled from a binomial distribution with a probability π that δ_jk_ was equal to 1 (i.e. the SNP has a non-zero effect) and a probability 1-π that δ_jk_ was equal to 0. The proportion 1-π was sampled from a beta distribution, B(α, β), with α = 300 and β = 29,652; the value of π was kept almost constant at 1%, corresponding to approximately 300 SNPs selected at each cycle from the 29,652 markers. This value of π was considered to be a good compromise in our variable selection algorithm between the high degree of polymorphism of the traits under study and the limited number of individuals (*n* = 1382) in our dataset.

The degree of association between each SNP and a given phenotype was assessed using the Bayes Factor (BF): $$ \mathrm{BF}=\frac{\raisebox{1ex}{${\mathrm{P}}_{\mathrm{i}}$}\!\left/ \!\raisebox{-1ex}{$\left(1-{\mathrm{P}}_{\mathrm{i}}\right)$}\right.}{\raisebox{1ex}{$\uppi $}\!\left/ \!\raisebox{-1ex}{$\left(1-\uppi \right)$}\right.} $$, where P_i_ is the probability that the i^th^ SNP has a non-zero effect.

Following Kass and Raftery (1995) [105], QTLs were evaluated based on calculations of 2*ln (BF) (twice the natural logarithm of the BF); the threshold 2*ln (BF) ≥ 6 was considered evidence for a QTL. As proposed by Michenet et al. (2016) [106], a credibility interval was constructed around the peak SNP that encompassed, within a sliding window of 1 Mb on either side, all neighbouring SNPs for which 2*ln (BF) ≥ 3. If 5 ≤ 2*ln (BF) < 6 for a peak, the QTL was considered putative, and was included in further analyses only if it explained at least 0.5% of the genetic variance for a trait or if it was linked with more than one trait.

All candidate genes that were located within the confidence or credibility intervals established using the BayesCπ approach are listed in Additional file 3, with annotation from the NCBI *Oncorhynchus mykiss* genome assembly release 100 (GCF_002163495.1), and gene symbols from Lallias et al. (2020) [107].

### Selection efficiency

To assess the relative efficiencies of a pedigree-based selection approach (best linear unbiased predictor; BLUP) and a genomic selection approach (GBLUP), estimated breeding values (EBV and GEBV, respectively) were derived via consideration of either the pedigree relationship matrix (A) or the genomic matrix (G) using the software package BLUPf90 [102].

For pedigree-based evaluation, the following BLUP model was applied for the estimation of breeding values:

$$ {Y}_i=\upmu +{Z}_i{\mathrm{a}}_i+{\mathrm{e}}_i $$

where Y_i_ is the performance of the i^th^ animal, μ is the overall mean of the population, and a_i_ and e_i_ are the vectors of additive genetic effects and residual effects that explain the performance of all phenotyped animals, respectively. Z is the incidence matrix for a_i_. In our model, vector a_i_ corresponded to the breeding values of 17,235 individuals related through the pedigree relationship matrix A of the 1382 phenotyped fish.

For genomic selection, the genomic relationship matrix G was used in place of the pedigree matrix A [108]. The following GBLUP model was applied for the estimation of genomic breeding values (GEBV):

$$ {Y}_i=\upmu +{Z}_i{\mathrm{g}}_i+{\mathrm{e}}_i $$

with the vector g_i_ corresponding to the breeding values of 1382 phenotyped and genotyped individuals related through the genomic relationship matrix G.

To assess the accuracy of (G) EBVs, 40 replicates of Monte Carlo ‘leave-one-group-out’ cross validation tests [109] were performed. For each replicate, 314 fish from the 1382 phenotyped and genotyped individuals were randomly chosen for the validation set and 1068 fish were chosen for the training set. The phenotypes recorded in the validation population were then masked and breeding values were estimated using (G) BLUP model.

For each replicate, the selection accuracy (Acc) was computed as:

$$ Acc=r\left((G){EBV}_v,y\right)/\surd h2 $$

where r represents the correlation between (G) EBV_*v*_ and y, (G) EBV_*v*_ represents the (genomic) breeding values of individuals belonging to the validation dataset, y is their phenotype, and h2 is the heritability estimated using pedigree information. In addition to evaluating selection accuracy, we also assessed the quality of BLUP and GBLUP evaluation by deriving the inflation coefficient of EBVs as a measure of selection bias. The inflation coefficient is the slope of the regression of the phenotypes on the (G)EBVs. In the absence of selection bias, this coefficient is expected to be equal to 1; in case of EBV over-dispersion (inflation), the coefficient is below 1 and in the case of EBV under-dispersion the value is above 1.

The values obtained for selection accuracy and the inflation coefficient were averaged over the 40 replicates.

## Supplementary Information

**Additional file 1.** Fatty acid composition of the two final diets (% total FAs).

**Additional file 2.** Estimates of phenotypic correlations between proportions of fatty acids and production or quality traits.

**Additional file 3.**Candidate genes from the NCBI *Oncorhynchus mykiss* Annotation Release 100 (GCF_002163495.1) that were located within the confidence or credibility intervals estimated using BCπ analysis.

**Additional file 4.** Summary statistics for 11 production and quality traits in rainbow trout.

## Data Availability

The data that support the findings of this study belong to the breeding company Aqualande; they were used under license for the current study, and so are not publicly available. The data can be made available for the purpose of reproduction of the results on request via a material transfer agreement and with the permission of Aqualande. Requests to access the datasets should be directed to Mathilde Dupont-Nivet or Vincent Petit.
