# Supplementary material for: Genetic architecture and genomic selection of fatty acid composition predicted by Raman spectroscopy in rainbow trout
Source: BMC Genomics. 2021 Nov 3;22:788. doi: 10.1186/s12864-021-08062-7 (PMC8564959; doi:10.1186/s12864-021-08062-7)
Supplement: Supplementary file 1 — Additional file 1. Fatty acid composition of the two final diets (% total FAs). [file 12864_2021_8062_MOESM1_ESM.docx]

**Additional file 1.** Fatty acid composition of the two final diets (% total FAs)

|  | **Viva Pro 7F NAT29** | **Extra CDC AQL G25** |
| --- | --- | --- |
| 12:0 | 0 | 0.146 |
| 14:0 | 1.486 | 3.425 |
| 15:0 | 0.175 | 0.199 |
| 16:0 | 9.752 | 20.429 |
| 17:0 | 0.164 | 0.138 |
| 18:0 | 1.971 | 2.127 |
| 20:0 | 0.285 | 0.211 |
| SFAs | 13.833 | 26.675 |
| 16:1 | 1.812 | 2.702 |
| 17:1 | 0.116 | 0 |
| 18:1 | 53.016 | 43.009 |
| 20:1 | 1.075 | 0.704 |
| 22:1 | 0.222 | 0.263 |
| MUFAs | 56.241 | 46.678 |
| 16:2 n-4 | 0 | 0.396 |
| 16:3 n-4 | 0.231 | 0.468 |
| 16:4 n-1 | 0.137 | 0.611 |
| 18:2 n-6 | 19.205 | 15.884 |
| 20:4 n-6 | 0.118 | 0.098 |
| PUFA n-6 | 19.323 | 15.982 |
| 18:3 n-3 | 7.411 | 5.688 |
| 18:4 n-3 | 0.308 | 0.513 |
| 20:5 n-3 | 0.949 | 2.258 |
| 22:6 n-3 | 1.154 | 0.720 |
| PUFA n-3 | 9.822 | 9.179 |
| LC PUFA n-3 | 2.103 | 2.978 |
