# Supplementary material for: Genetic architecture and genomic selection of fatty acid composition predicted by Raman spectroscopy in rainbow trout
Source: BMC Genomics. 2021 Nov 3;22:788. doi: 10.1186/s12864-021-08062-7 (PMC8564959; doi:10.1186/s12864-021-08062-7)
Supplement: Supplementary file 2 — Additional file 2. Estimates of phenotypic correlations between proportions of fatty acids and production or quality traits. [file 12864_2021_8062_MOESM2_ESM.docx]

|  | BW | Fat | K | MRI_F_sc% | MRI_F% | MRI_F_F% | L_flesh | a_flesh | b_flesh | HGCarc% | Carc% |
| --- | --- | --- | --- | --- | --- | --- | --- | --- | --- | --- | --- |
| SFA | 0.16 (±0.03) | 0.14 (±0.03) | 0.13 (±0.03) | 0.11 (±0.03) | 0.16 (±0.03) | 0.16 (±0.03) | -0.03 (±0.03) | 0.14 (±0.03) | 0.15 (±0.03) | -0.01 (±0.03) | -0.07 (±0.03) |
| MUFA | 0.24 (±0.03) | 0.21 (±0.03) | 0.18 (±0.03) | 0.2 (±0.03) | 0.27 (±0.03) | 0.21 (±0.03) | 0 (±0.03) | 0.21 (±0.03) | 0.14 (±0.03) | -0.05 (±0.03) | -0.17 (±0.03) |
| PUFA | -0.25 (±0.03) | -0.21 (±0.03) | -0.2 (±0.03) | -0.19 (±0.03) | -0.27 (±0.03) | -0.24 (±0.03) | 0.02 (±0.03) | -0.23 (±0.03) | -0.19 (±0.03) | 0.04 (±0.03) | 0.15 (±0.03) |
| n-3 PUFA | -0.11 (±0.03) | -0.11 (±0.03) | -0.11 (±0.03) | -0.11 (±0.03) | -0.14 (±0.03) | -0.12 (±0.03) | 0.04 (±0.03) | -0.1 (±0.03) | -0.11 (±0.03) | 0.01 (±0.03) | 0.04 (±0.03) |
| n-6 PUFA | -0.37 (±0.03) | -0.26 (±0.03) | -0.25 (±0.03) | -0.21 (±0.03) | -0.32 (±0.03) | -0.3 (±0.03) | -0.03 (±0.03) | -0.31 (±0.03) | -0.2 (±0.03) | 0.08 (±0.03) | 0.28 (±0.03) |
| OA | 0.12 (±0.03) | 0.11 (±0.03) | 0.1 (±0.03) | 0.13 (±0.03) | 0.17 (±0.03) | 0.13 (±0.03) | 0.03 (±0.03) | 0.1 (±0.03) | 0.05 (±0.03) | -0.06 (±0.03) | -0.12 (±0.03) |
| LA | -0.31 (±0.03) | -0.22 (±0.03) | -0.22 (±0.03) | -0.18 (±0.03) | -0.28 (±0.03) | -0.26 (±0.03) | -0.03 (±0.03) | -0.26 (±0.03) | -0.16 (±0.03) | 0.08 (±0.03) | 0.25 (±0.03) |
| ALA | -0.03 (±0.03) | -0.03 (±0.03) | -0.03 (±0.02) | -0.02 (±0.03) | -0.02 (±0.03) | -0.04 (±0.03) | 0.05 (±0.03) | -0.02 (±0.03) | -0.06 (±0.03) | -0.06 (±0.03) | -0.06 (±0.03) |
| ARA | -0.28 (±0.03) | -0.24 (±0.03) | -0.19 (± 0.03) | -0.21 (±0.03) | -0.29 (±0.03) | -0.24 (±0.03) | 0 (±0.03) | -0.27 (±0.03) | -0.19 (±0.03) | 0.04 (±0.03) | 0.16 (±0.03) |
| EPA | -0.25 (±0.03) | -0.2 (±0.03) | -0.18 (± 0.03) | -0.17 (±0.03) | -0.25 (±0.03) | -0.21 (±0.03) | -0.03 (±0.03) | -0.21 (±0.03) | -0.14 (±0.03) | 0.06 (±0.03) | 0.18 (±0.03) |
| DHA | -0.1 (±0.03) | -0.09 (±0.03) | -0.1 (±0.03) | -0.13 (±0.03) | -0.16 (±0.03) | -0.12 (±0.03) | -0.03 (±0.03) | -0.09 (±0.03) | -0.07 (±0.03) | 0.08 (±0.03) | 0.12 (±0.03) |
| EPA+DHA | -0.13 (±0.03) | -0.11 (±0.03) | -0.11 (±0.03) | -0.13 (±0.03) | -0.18 (±0.03) | -0.13 (±0.03) | -0.03 (±0.03) | -0.11 (±0.03) | -0.08 (±0.03) | 0.07 (±0.03) | 0.12 (±0.03) |

**Additional file 2.** Estimates of phenotypic correlations between proportions of fatty acids and production or quality traits

(BW = Body weight; Fat = Percentage of fat with Fatmeter, K = Fulton coefficient, MRI_F_sc% = Percentage of subcutaneous fat in whole steak using MRI, MRI_F%= Percentage of fat in whole steak using MRI, MRI_F_F% = Percentage of fat flesh in whole steak using MRI, L_flesh = Flesh colour luminosity, a_flesh = Flesh redness, b_flesh = Flesh yellowness, HGCarc% = Headless gutted carcass yield, Carc% = Carcass yield)
