# Supplementary material for: Genetic architecture and genomic selection of fatty acid composition predicted by Raman spectroscopy in rainbow trout
Source: BMC Genomics. 2021 Nov 3;22:788. doi: 10.1186/s12864-021-08062-7 (PMC8564959; doi:10.1186/s12864-021-08062-7)
Supplement: Supplementary file 4 — Additional file 4. Summary statistics for 11 production and quality traits in rainbow trout. [file 12864_2021_8062_MOESM4_ESM.docx]

**Additional file 4.** Summary statistics for 11 production and quality traits in rainbow trout

| Name | Trait | N | Mean | SD | Min | Max | CV (%) |  |
| --- | --- | --- | --- | --- | --- | --- | --- | --- |
|  |  |  |  |  |  |  |  |  |
| BW | Body weight (g) | 1382 | 2139.24 | 460.98 | 658 | 3363 | 21.55 |  |
| Fat | %Fat with Fatmeter | 1380 | 13.88 | 2.56 | 4.3 | 20.3 | 18.45 |  |
| K | Fulton coefficient K = (BW(g) ∗ 100/BL3 (cm)) | 1382 | 1.38 | 0.12 | 0.99 | 1.80 | 8.91 |  |
| MRI_F_sc% | Percentage of subcutaneous fat in whole steak with MRI technology | 1284 | 27.00 | 2.71 | 16.42 | 37.74 | 10.03 |  |
| MRI_F% | Percentage of fat in whole steak with MRI technology | 1285 | 26.22 | 2.99 | 16.01 | 34.84 | 11.43 |  |
| MRI_F_F% | Percentage of fat flesh in whole steak with MRI technology | 1285 | 12.53 | 2.00 | 6.61 | 19.83 | 15.99 |  |
| L_flesh | Flesh colour Luminosity | 1382 | 49.61 | 1.50 | 44.90 | 55.65 | 3.03 |  |
| a_flesh | Flesh colour a redness | 1382 | 35.68 | 1.78 | 27.23 | 40.38 | 4.99 |  |
| b_flesh | Flesh colour b yellowness | 1382 | 43.71 | 1.76 | 31.48 | 49.96 | 4.02 |  |
| HGCarc% | Headless gutted carcass yield (HGCarc% = (BW - (Head weight + viscera weight)) / BW*100 ) | 1378 | 80.64 | 1.27 | 75.36 | 84.63 | 1.57 |  |
| Carc% | Carcass yield (Carc% = (BW - viscera weight)/ BW*100 | 1378 | 91.48 | 1.14 | 87.19 | 94.87 | 1.24 |  |

(N = number of individuals; SD = standard deviation; Min = minimum value; max = maximum value; CV = coefficient of variation (SD/mean*100))
